# Memory deficits in hypertensive ApoE4 mice reversed by P2Y12 inhibition via different mechanisms in males and perimenopausal females

**DOI:** 10.21203/rs.3.rs-7643285/v1

**Published:** 2025-09-26

**Authors:** Lianne Trigiani, Nicole Chernavsky, Rachel Kim, Nuri Hong, Robert Hawkins, Emily Le, Zahra Bahninameh, Keri Yamaguchi, Jonah Bernard, Amanda Huang, Daniel Rivera, Nathaniel Allan-Rahill, Michael Lamont, Roberta Marongiu, Costantino Iadecola, Nozomi Nishimura, Chris Schaffer

**Affiliations:** Nancy E. and Peter C. Meinig School of Biomedical Engineering; Nancy E. and Peter C. Meinig School of Biomedical Engineering; Nancy E. and Peter C. Meinig School of Biomedical Engineering; Nancy E. and Peter C. Meinig School of Biomedical Engineering; Nancy E. and Peter C. Meinig School of Biomedical Engineering; Nancy E. and Peter C. Meinig School of Biomedical Engineering; Nancy E. and Peter C. Meinig School of Biomedical Engineering; Cornell University; Nancy E. and Peter C. Meinig School of Biomedical Engineering; Nancy E. and Peter C. Meinig School of Biomedical Engineering; Cornell University; Nancy E. and Peter C. Meinig School of Biomedical Engineering; Nancy E. and Peter C. Meinig School of Biomedical Engineering; Weill Cornell Medicine; Weill Cornell Medicine; Cornell University; Cornell University

## Abstract

Apolipoprotein E4 (ApoE4) genotype, hypertension, and biological sex are critical risk factors for Alzheimer’s disease and related dementias. Yet, their combined impact on early cerebrovascular dysfunction, brain inflammation, and memory impairment remains poorly understood. We developed a translational mouse model incorporating human ApoE4, hypertension via angiotensin II infusion, and induced accelerated ovarian failure (AOF) to mimic perimenopause in females to investigate these interactions. Hypertensive ApoE4 mice of both sexes exhibited impaired spatial working memory, decreased cerebral blood flow, increased neuroinflammation, and decreased blood brain barrier integrity, recapitulating key early clinical features observed in human populations with these risk factors. Brain blood flow reduction was associated with an increased incidence of capillary stalling, with notable sex differences in the extent and cellular composition of stalls: in males, stalling was strongly elevated and mostly due to red blood cell arrest, while stalling was modestly elevated in peri-AOF females with most stalls including leukocytes. Treatment with prasugrel, a P2Y12 receptor inhibitor, improved memory performance in both sexes but was correlated with different physiological effects – restored cerebral blood flow in males and reduced microglia motility and inflammation in peri-AOF females. Platelet depletion mimicked prasugrel’s blood flow and cognitive benefits in males, while microglia depletion selectively rescued memory in females. Our work emphasizes the necessity of including translationally relevant female mouse models in neurodegenerative disease studies, and our findings highlight the importance of risk profile-specific interventions and demonstrate that early vascular dysfunction may be a key, sex-dependent driver of cognitive decline.

## One Sentence Summary

ApoE genotype, hypertension, female sex, and perimenopause interact to impair memory via sex-specific vascular and inflammatory mechanisms that are reversible by P2Y12 inhibition.

## INTRODUCTION

Deficits in cerebrovascular function, including reduced vascular reactivity, cerebral perfusion deficits, and increased blood brain barrier (BBB) permeability, are identified as early events in the development of Alzheimer’s disease (AD) in humans^1–3^, and may have even greater relevance for AD-related dementias (ADRD) that often include significant vascular contributions. There are several genetic and cardiovascular risk factors for ADRD, including Apolipoprotein E (ApoE) genotype, biological sex, menopause, and hypertension, that have also been tied to cerebrovascular alterations. Carrying the ε4 allele of ApoE (ApoE4) is the strongest and most prevalent genetic risk factor for late onset AD, conferring a 4- to 12-fold increase in risk and present in 14–20% of the population^4,5^. Disruption in cerebrovascular function has been noted throughout the lifespan in ApoE4 carriers^6–9^, with region-specific brain hyperperfusion in midlife and a more rapid decline in perfusion later in life, as compared to ApoE3 carriers. Additionally, in ApoE4 carriers this age-related perfusion decline is faster in women^10^.

The prevalence of late onset AD is also much higher in women than men, representing 65% of cases globally, even when matched for cardiovascular risk profiles^11,12^. This discrepancy is potentially attributable to the transition to menopause, which is often not accounted for in patients and even less so in animal models. A frequently reported symptom during this transition is that of “brain fog”, a feeling of reduced mental clarity that does not rise to clinical significance^13,14^. Recently, the menopause transition (or perimenopause) in cognitively normal women has been associated with cerebral hypometabolism, elevated levels of oxidative cellular damage, and decreased cerebral blood flow (CBF) and cerebrovascular reactivity^15,16^. Another prominent and prevalent cardiovascular risk factor that has been associated with ApoE4 status and increased AD risk is hypertension^17,18^, with a recent study reporting that 82% of individuals with a dementia diagnosis present with high blood pressure, compared to only 32% of cognitively normal individuals^19^. In a longitudinal study, a 9% decrease in a measure of cognitive abilities was observed for every 10 mmHg increase in blood pressure that was measured 20 years prior to cognitive testing^20^. In ApoE4 carriers, elevated blood pressure has been found to drive greater cognitive impairment^21^, even in the absence of diagnosed dementia^22–24^. Studies in cognitively normal individuals have reported decreased CBF due to elevated systolic blood pressure, which can be mitigated by treatment with antihypertensive medications^25–27^. While the risk of hypertension is more commonly associated with men, women are equally likely to be diagnosed with high blood pressure by their sixth decade of life, after menopause^28^, and with every 10 mmHg increase in systolic blood pressure post-menopausal women experience a 25% increased risk for cardiovascular disease relative to a 15% increase in age-matched men^29^. Studying the interaction mechanisms of potent drivers of ADRD including ApoE4, hypertension, biological sex, and menopause could thus aid in our understanding of how cognitive decline emerges.

While it is becoming increasingly accepted that reduced cerebral perfusion is an early occurrence in ADRD pathogenesis^1,30,31^, the molecular and cellular mechanisms underlying this deficit remain in the early stages of investigation. Capillary stalling has been identified as a potential mechanism contributing to this CBF deficit, wherein a small percentage (1–2%) of the brain’s microcirculation is transiently plugged^32^. We previously showed this capillary stalling accounts for most of the ~20–30% CBF reduction in the APP/PS1 mouse model of AD^32,33^. Other researchers have similarly shown that capillary stalling drives CBF deficits in the APP^NL-G-F^ model of AD^34^, and in a mouse model of type 1 diabetes^35^. However, whether capillary stalling contributes to CBF reductions secondary to ApoE allele status or hypertension, as well as how this might be impacted by female sex and menopause, has not yet been investigated.

In this paper, we developed a new mouse model of ADRD by combining highly prevalent risk factors (ApoE genotype, hypertension, and biological sex – including the effects of perimenopause) and observed a pattern of memory impairment resembling that of patients with these risk factors. To achieve this, we used mice with a targeted replacement of murine ApoE with the human ε3 or ε4 allele (ApoE3-TR or ApoE4-TR mice, respectively) challenged with a slow pressor dose of angiotensin II (AngII). In female mice, estrogen (one of two ovarian hormones for which levels decline with menopause) is protective against AngII-induced hypertension^36^, but by driving ovarian failure and placing them in a perimenopause-like state, we rendered females sensitive to the effects of AngII. We aimed to see how these risk factors might interact to impact CBF, neuropathology, and memory function. We found an impairment of cognitive function uniquely in hypertensive ApoE4-TR mice, accompanied by a reduction in CBF and an increase in the frequency of capillary stalls, similar to our prior findings in amyloid models, albeit with a more diverse cellular composition of stalls. Additionally, disruptions in BBB integrity and mild increases in neuroinflammation were associated with ApoE4 and hypertension, with some sex-dependent differences in microglia behaviors. Finally, we found that treatment with the irreversible P2Y12 receptor inhibitor prasugrel improved spatial working memory in hypertensive ApoE4 mice of both sexes. Prasugrel treatment improved BBB integrity in both sexes with sex-dependent effects of prasugrel on CBF and neuroinflammation. Studies where we depleted circulating platelets or brain-resident microglia showed sexually dimorphic rescue of memory function that paralleled the sex-dependent effects of prasugrel on capillary stalling, CBF, and neuroinflammation. This study emphasizes the need for targeted therapeutic approaches that consider individual risk profiles, and it emphasizes the importance of female mice being studied

## MATERIALS AND METHODS

### Study design

All animal procedures were approved by Cornell University’s Institutional Animal Care and Use Committee (Protocol #: 2015-0029). Cornell University has an approved Animal Welfare Assurance protocol with the Office of Laboratory Animal Welfare (Assurance #: D16-00225) and is accredited by the Association for Assessment and Accreditation of Laboratory Animal Care International. All experiments conform to these guidelines. Experiments were performed in homozygous ApoE3-TR and ApoE4-TR mice on a C57BL/6 genetic background^102^, in males and females where we pharmacologically induced accelerated ovarian failure (AOF) to place mice in a perimenopause-like state (peri-AOF females). To allow imaging of microglia in a subset of experiments, ApoE3-TR and ApoE4-TR were crossed with CX_3_CR-1^GFP^ mice^103^ and back-crossed until homozygous for their respective ApoE allele, henceforth referred to as Cx3cr1-ApoE3 or Cx3cr1-ApoE4, respectively. At the time that imaging experiments began, mice were between 5–6 months of age. Animals were group housed, with a 12-h light cycle. Detailed descriptions of experimental methods, mouse treatments, data analyses, and statistical approaches are in the supplementary material, including tables with details of all statistical tests included in the main figures (Supplementary table 1) and the supplementary figures (Supplementary table 2). We use a standardized set of significance indicators in all plots: *p < 0.05, **p < 0.01, ***p < 0.001.

Briefly, four separate cohorts of mice underwent longitudinal *in vivo* brain imaging and behavioral studies, whereby the same mice were assessed at multiple timepoints. In the first cohort of mice, we investigated how ApoE genotype, hypertension, and female sex impacted cognition and cerebrovascular function at baseline and after two weeks of AngII-induced hypertension (via implanted osmotic minipump) in ApoE3-TR and ApoE4-TR males as well as females with and without AOF. To focus on potential mechanisms contributing to the memory impairment we found in hypertensive male and peri-AOF female ApoE4-TR mice alone, we only included these two groups in the next three cohorts. In the second cohort, which evaluated the impact of prasugrel treatment, mice were assessed at baseline, after two weeks of induced hypertension, and either shortly after a single dose of prasugrel (with *in vivo* imaging measures acquired ~30 minutes after treatment and behavioral measures acquired 48 hours after treatment) or after seven days of prasugrel treatment (each with continued hypertension). We also evaluated a variety of histopathological and hematological outcomes in the first two cohorts. The last two cohorts of mice aimed to evaluate potential mechanisms contributing to the memory improvement we observed with prasugrel treatment by depleting platelets from the blood (cohort 3) or microglia from the brain (cohort 4). These mice were evaluated after two weeks of induced hypertension, and again 24 hours following platelet depletion or seven days following microglia depletion (with continued hypertension in both cases).

### Statistics

For experiments with repeated measures using ApoE3-TR and ApoE4-TR mice, repeated measures two-way ANOVAs were used for all *in vivo* data, followed by Šídák’s multiple comparisons test between genotypes and timepoints. For experiments in ApoE4-TR mice measured at multiple timepoints, one-way repeated measures ANOVAs were performed. In all cases, mixed-effects ANOVAs were used in the case of any missing values due to degrading quality of cranial windows or inability to relocate the same vessel at multiple timepoints. In instances where values are represented as a fraction of baseline, paired t-tests were performed. For cumulative frequency plots, a Kolmogorov-Smirnov test was performed. For all analyses, outliers were excluded if they exceeded the group mean ± three times the standard deviation. P-values less than 0.05 were considered statistically significant and are denoted throughout the figures as: *p < 0.05, **p < 0.01, ***p < 0.001. GraphPad Prism 10 software was used for all statistical analyses and plotting.Box and whisker plots display the median, interquartile range between the 25^th^ and 75^th^ percentile, and whiskers extending to the maximum and minimum. See supplementary Tables 1 and 2 for details on the individual statistical tests performed for each comparison shown in the main and supplementary figures, respectively.

## RESULTS

### Hypertension caused a decline in spatial working memory exclusively in ApoE4-TR mice

**Figure 1:**
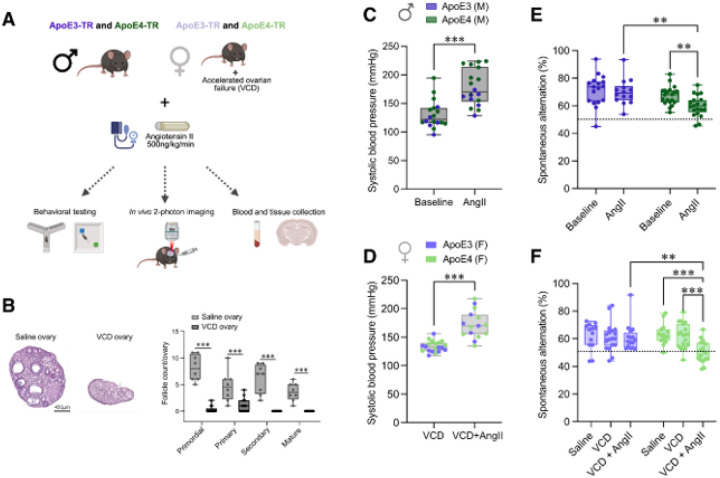
Hypertension caused a decline in spatial working memory exclusively in ApoE4-TR mice. (**A**) Mouse groups with their respective risk factors and outcome measures. (**B**) Hematoxylin and Eosin staining of ovaries (*left*) for follicle counts (*right*) in female mice treated with saline or vinylcyclohexene diepoxide (VCD) harvested after the last *in vivo* imaging session. (**C-D**) Systolic blood pressure measured in awake mice via tail cuff at baseline and after two weeks of subcutaneous angiotensin II (AngII) infusion (500 ng/kg/min) in males (C) and VCD-females (D). (**E-F**) Spontaneous alternation scores from a 6-min Y-maze task to assess spatial working memory in males (E) and in females (F).

In this study we aimed to capture early pathological and pathophysiological features that correlated with memory deficits in a novel murine ADRD model based on prevalent risk factors found in patients, using both *in vivo* and *ex vivo* approaches (Fig. 1A). We evaluated the impact of three important risk factors – ApoE genotype, hypertension, and female sex with peri-AOF. Because female mice are protected by estrogen from AngII-induced hypertension^37,38^, we rendered them sensitive to AngII by inducing a perimenopause-like state using 4-vinylcyclohexene diepoxide (VCD) injections to drive accelerated ovarian failure (VCD-female), a model which more closely recapitulates the transition to menopause seen in humans^39^. We also included an additional group of age-matched female mice with intact ovaries to control for any potential consequences of peri-AOF alone in the VCD-treated groups. To verify ovarian failure, ovaries were collected from females at the study endpoint. VCD-injected mice had a paucity of growing follicles and reduced ovary size relative to age-matched intact pre-menopausal mice (Fig. 1B, *left*). Further evaluating a subset of VCD-female mice, we found a strong decrease in the number of follicles at each developmental stage (Fig. 1B, *right*), but with no elevation in follicular stimulating hormone (FSH) (Supplementary fig. 1), suggesting the VCD-female mice in our studies were within a perimenopausal-like phase.

Mean, systolic, and diastolic blood pressure increased in male (Fig. 1C, Supplementary fig. 2A) and VCD-female (Fig. 1D, Supplementary fig. 2B) mice after two weeks of AngII administration. Reduced spontaneous alternation on the Y-maze task was observed exclusively in hypertensive ApoE4-TR males (Fig. 1E, *p* < 0.01) and VCD-females (Fig. 1F, *p* < 0.001), as compared to normotensive ApoE4-TR or hypertensive ApoE3-TR counterparts, indicative of worse spatial working memory. Peri-AOF alone did not impact performance in the spontaneous alternation task (Fig. 1F). No significant differences emerged between groups in object recognition memory on the novel object recognition task, although ApoE4-TR mice, independent of female sex or hypertension, tended toward reduced novel object recognition as measured by investigation ratio, as compared to ApoE3-TR (Supplementary fig. 3).

### Pathological features of ADRD in hypertensive ApoE4-TR mice

**Figure 2:**
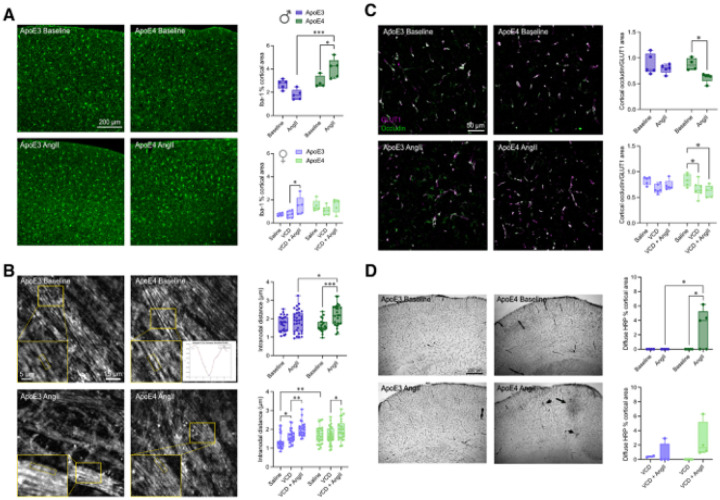
Hypertension worsens pathological features of ADRD with more severe impacts in ApoE4-TR mice. (**A**) Immunohistology of Iba-1-positive cortical microglia in males and females. (**B**) *In vivo* THG images of sub-cortical myelin. Insets highlight putative nodes of Ranvier, while one inset shows a Gaussian fit to extract the intranodal distances shown in plots. (**C**) Cortical occludin area coverage (normalized to GLUT1 area) in males and females. (**D**) Blood brain barrier disruption with horseradish peroxidase (HRP) leakage from vasculature (black arrows) in hypertensive ApoE4-TR mice.

Although a focus of this study is on cerebrovascular consequences of the examined risk factors, we also assessed astrocyte and microglia reactivity, changes in sub-cortical myelin using *in vivo* third harmonic generation (THG) microscopy, as well as BBB integrity by immunohistochemistry. Hypertension was associated with modest increases in neuroinflammation, with trends towards increased area covered by reactive astrocytes (GFAP) in males regardless of genotype (Supplementary fig. 4A-B), and significant increases in Iba-1-positive microglia area in ApoE4-TR males (*p* < 0.05) and ApoE3-TR VCD-females (*p* < 0.05) with hypertension (Fig. 2A). We found no notable changes in microglia branching morphology between conditions or genotype in males (Supplementary fig. 5A). On the other hand, in VCD-females, microglial processes showed greater ramification and complexity of cellular process morphology in ApoE4-TR, as compared to ApoE3-TR mice, and this difference persisted with AngII administration (Supplementary fig. 5B). Additionally, ApoE4-TR VCD-females had increased microglia branch length associated with hypertension.

To assess early signs of white matter disruption, intranodal distances of putative nodes of Ranvier were measured in the corpus callosum using *in vivo* THG imaging^40^ (Fig. 2B). In females at baseline (no VCD injections), we found increased intranodal distance with ApoE4 genotype, as compared to ApoE3 (p < 0.01). In female ApoE3 mice, driving peri-AOF increased intranodal distance and hypertension increased it further (p < 0.01), while in female ApoE4-TR mice, no increase was observed after VCD injection, but hypertension did drive an increase in the ApoE4-TR VCD-females (*p* < 0.05) (Fig. 2B). In male ApoE4 mice, AngII-induced hypertension increased intranodal distance (p < 0.001), while hypertension did not have an effect in ApoE3 males (Fig. 2B).

In males, we observed a compromise of BBB integrity uniquely in hypertensive ApoE4-TR mice, as evidenced by decreased occludin coverage of brain capillaries (Fig. 2C, *p* < 0.05), increased levels of vascular matrix metalloproteinase-9 (MMP9) (Supplementary fig. 6A, *p* < 0.01), and increased horseradish peroxidase (HRP) extravasation, a marker of more severe BBB compromise (Fig. 2D, *p* < 0.05). In ApoE4 females, we observed indicators of compromised BBB integrity due to VCD injection that were not exacerbated by hypertension, including decreased occludin coverage in both normotensive and hypertensive VCD-females (Fig. 2C, *p* < 0.05), and a trend toward increased vascular MMP9 (Supplementary fig. 6B, *p* = 0.11). Increased HRP extravasation was observed only with hypertension in ApoE4 VCD-female mice (Fig. 2D). Both normotensive and hypertensive ApoE3 VCD-female mice showed a trend toward decreased occludin coverage (*p* = 0.07), slight extravasation of HRP, but no clear changes in vascular MMP9, suggesting a more subtle change in BBB integrity than in ApoE4 VCD-females.

To further characterize our ADRD model and gain insights into changes in peripheral and central inflammatory status, we examined markers of platelet activation from peripheral blood, and quantified 62 cytokines from plasma and cortical samples. We measured platelet activation status using fluorescent activated cell sorting on whole blood samples to label activated platelets with CD62P (P-selectin) and JON/A (activated status of platelet GPIIb/IIIa receptor), as well as platelet-leukocyte aggregates with the neutrophil marker anti-Ly6G (Supplementary fig. 7). Regardless of genotype or female sex, hypertension increased markers of platelet activation: CD62P (*p* < 0.05 in males and VCD-females) and JON/A (*p* < 0.05 in males, *p* < 0.01, in females). In plasma, males and VCD-females showed similar patterns of cytokine expression at baseline, with no significant genotype differences observed in males, while in VCD-females several cytokines exhibited statistically significant increases with ApoE4, as compared to ApoE3, but all with relatively modest effect sizes. With hypertension, several cytokines were significantly up- or down-regulated, with ~2.5 times more cytokines significantly changed in VCD-females, as compared to males. For example, hypertension increased levels of insulin-like growth factor binding protein-3 (IGFBP-3) and leptin R in both sexes, while platelet factor 4 (PF4) was decreased only in females (Supplementary fig. 8C-D). In males, hypertension-induced changes did not differ between genotypes. However, in VCD-females, there were significantly reduced levels of fractalkine and monocyte chemoattractant protein 1 (MCP-1) with hypertension in ApoE4 as compared to ApoE3 mice (Supplementary fig. 8B). Cortical samples were analyzed only in ApoE4-TR groups (Supplementary fig. 9). As with the plasma samples, there were generally more changes (2-fold greater) in VCD-females with hypertension, relative to males. We saw similar decreases with hypertension in males and VCD-females for Axl, IGFBP-5, IL-6, IL-10, IL-13, and Leptin, together with an increase in IL-1α. In a principal component (PC) analysis of the plasma (Supplementary fig. 8G) and cortex (Supplementary fig. 9E) cytokine data, we found clear, separate clusters for the baseline and AngII-treated mice. For the plasma samples, there did not appear to be differences by genotype or sex in this clustering, while for the cortical samples there seemed to be larger changes in females (along PC1) associated with hypertension. These observations collectively suggest a sex-dependent divergence in inflammatory response to hypertension, with VCD-ApoE4-females exhibiting the most pronounced changes in the cortex.

### Hypertension increased capillary stalling in ApoE4-TR mice with sex-specific differences in the cellular composition of stalls

**Figure 3:**
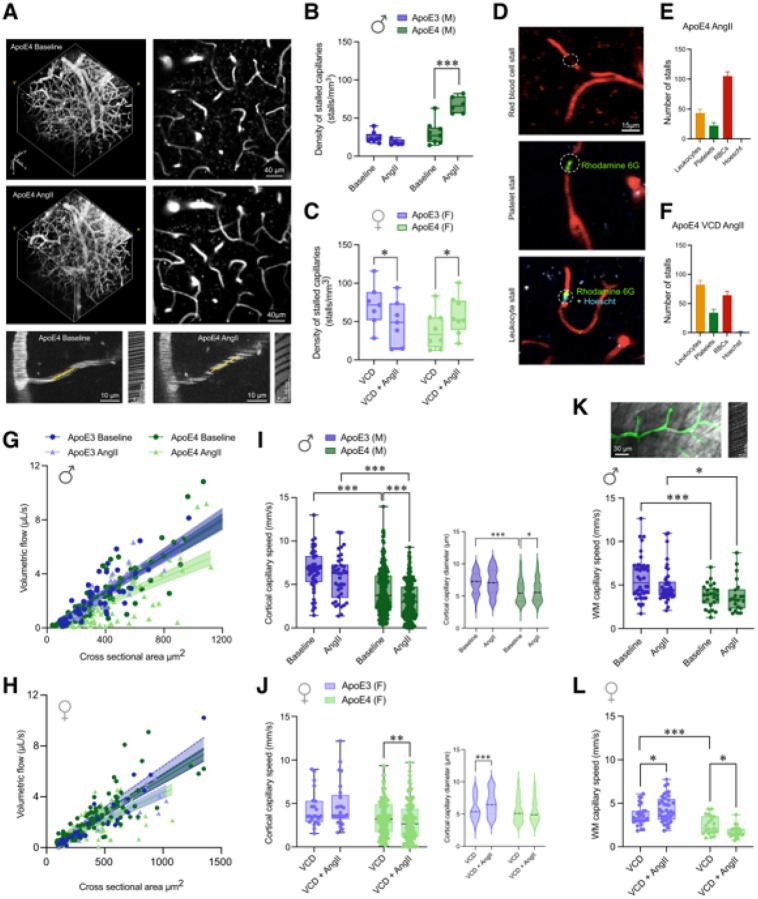
Hypertension decreased CBF and increased capillary stalling in ApoE4-TR mice. (**A**) 2PEF images and line-scans before and after AngII (2 weeks). (**B**) Capillary stall incidence in males and (**C**) VCD-females. (**D-F**) Stall composition (RBCs, platelets, or leukocytes) in hypertensive ApoE4-TR males (E, *n* = 6, 170 stalls) and VCD-females (F, *n* = 8, 181 stalls). Colors in D reflect plasma (red; Texas Red dextran), platelets (green; rhodamine 6G), or leukocytes (blue-green overlap; rhodamine 6G and Hoechst). Error bars indicate binomial confidence intervals. Volumetric flow as a function of vessel cross sectional area in penetrating arterioles in males (**G**) and VCD-females (**H**); data fitted using linear regression. Cortical capillary speed and diameter in males (**I**), and VCD-females (**J**). 3PEF quantification of capillary flow speeds in white matter in males (**K**), and VCD-females (**L**).

To assess microvascular changes, we first examined the dynamic process of capillary stalling, measuring the density of stalled capillaries and characterizing the cellular composition within them using *in vivo* two-photon excitation fluorescence (2PEF) imaging (Fig. 3A).^41^ Since we did not observe spatial working memory deficits attributable to peri-AOF alone in this model, all subsequent experiments in females used only VCD-injected mice. We found that hypertension increased the number of stalled capillary segments measured in the same tissue volume only in ApoE4-TR male (2.2-fold increase, *p* < 0.001, Fig. 3B) and ApoE4-TR VCD-female mice (1.5-fold increase, *p* < 0.05, Fig. 3C). We also observed an unexpected, small decrease in stall incidence in ApoE3-TR VCD-female mice with hypertension (*p* < 0.05). Cellular components located within the stalled segments differed by sex (Fig. 3D). Stalls containing only red blood cells (RBCs) dominated in hypertensive ApoE4-TR males (Fig. 3E), while stalls in hypertensive ApoE4 VCD-females contained more leukocytes (Fig. 3F). To explore the role of focal vessel diameter constrictions in causing these capillary stalls, we labeled the vessel wall with fluorescently labeled WGA-lectin in a subset of hypertensive ApoE4-TR male mice. We found 90 stalls in this subset, 27% of which had a clear focal constriction adjacent to the arrested blood cell, with a 30±4% average decrease in diameter (1.0±0.1 μm) relative to the rest of the stalled segment (Supplementary fig. 10). The remaining 73% of vessels showed a modest increase in vessel diameter (15±1% increase or 0.7±0.1 μm) at the site of the arrested blood cell.

### Hypertension decreased cerebral blood flow in ApoE4-TR mice

We used 2PEF imaging to measure RBC flow velocity in cerebral penetrating arterioles and capillaries within the top 300 μm of the cerebral cortex, and three-photon excited fluorescence (3PEF) imaging to measure deeper capillaries within the white matter of the corpus callosum. In the cortex of both male and VCD-female ApoE4-TR mice, we found that blood flow was consistently slower after AngII infusion, relative to baseline (Fig. 3G–J). In penetrating arterioles, this decreased flow is indicated by a reduced slope in the relationship between volumetric blood flow and cross-sectional area, indicating that vessels of the same size have less volumetric flow. In ApoE4-TR mice, we observed reduced flow in both male (Fig. 3G, *p* < 0.01) and VCD-female mice (Fig. 3H, *p* < 0.001) after AngII infusion as compared to baseline. In addition, ApoE3-TR VCD-female mice showed decreased penetrating arteriole flow after inducing hypertension (Fig. 3H, *p* < 0.05), while hypertension did not decrease flow speed in ApoE3-TR male mice. Evaluating just centerline penetrating arteriole flow velocity, without adjusting for vessel diameter, we found a decrease due to AngII in both genotypes, but with larger effects in ApoE4-TR mice. ApoE4-TR males had a 40% decrease in velocity (9.7±0.4 vs. 5.8±0.7 mm/s, *p* < 0.001), while ApoE4-TR VCD-females had a 15% decrease (8.5±0.5 vs. 7.2±0.4 mm/s, *p* < 0.01). In response to the same AngII challenge, ApoE3-TR males showed a 5% decrease (13.0±0.8 vs. 12.2±0.7 mm/s, *p* < 0.05), while ApoE3-TR VCD-females showed a 10% decrease (8.7±0.7 vs. 7.9±0.5 mm/s, *p* < 0.05). We did not observe any difference in diameter of penetrating arterioles at baseline and after inducing hypertension in either genotype in males or VCD-females (Supplementary fig. 11).

We observed similar trends in capillaries. In both the cortex and white matter (WM) of males, we observed that ApoE4-TR capillaries had slower flow rates compared to ApoE3-TR, independent of hypertension (Fig. 3I and K, *p* < 0.001). We also observed slower flow in the WM of ApoE4-TR VCD-females as compared to ApoE3-TR mice (Fig. 3L, *p* < 0.001). It is important to note that there were some modest, but significant, differences in capillary diameter between genotypes, as the randomly sampled capillaries in ApoE4-TR males were smaller than those measured in ApoE3-TR mice in the cortex (Fig. 3I *right*), while the opposite was true for VCD-female capillaries in the WM, warranting some caution in the interpretation of these flow speed comparisons across genotypes. We further found that hypertension significantly decreased capillary flow speeds in ApoE4-TR males and VCD-females when compared to baseline. Despite a slight increase in capillary diameters in ApoE4-TR males after AngII (Fig. 3I, *right*, *p* < 0.05), there was a 25% decrease in flow speed relative to baseline (4.2±0.2 vs. 3.2±0.2 mm/s, *p* < 0.001). Capillary flow in ApoE4-TR VCD-females showed a similar pattern to that observed in males, with a 13% decrease relative to baseline (3.37±0.20 vs. 2.93±0.21 mm/s, *p* < 0.01, Fig. 3J, *left*). In the cortex, hypertensive ApoE3-TR males showed a trend towards decreased capillary flow speeds, while in ApoE3-TR VCD-females, there was a surprising trend towards increased capillary flow with hypertension that was accompanied by an increase in capillary diameter (Fig. 3J, *right*, *p* < 0.001). In WM capillaries, flow speed was not worsened by the addition of hypertension in males. In contrast, VCD-females showed a significant decrease after hypertension exclusively in ApoE4-TR females (*p* < 0.05), and the same surprising increase in flow speed in ApoE3-TR females with hypertension (*p* < 0.05, Fig. 3L).

Finally, we used multi-exposure speckle contrast analysis imaging to assess cortical perfusion (Supplementary fig. 12) and further confirmed these results. In ApoE4-TR males cortical perfusion decreased by 31% due to hypertension (*p* < 0.05), while ApoE3-TR males showed a trend towards reduced perfusion. In VCD-females, we noted a 37% decrease in perfusion in ApoE4-TR mice (p<0.05) and a trend toward increased flow in ApoE3-TR mice. As with our 2PEF results, these data indicate that hypertension significantly impairs cortical perfusion, with the effect depending on both sex and ApoE allele status.

### Improved memory function and sex-dependent reversal of cerebral pathologies by acute and chronic P2Y12 receptor inhibition with prasugrel

**Figure 4:**
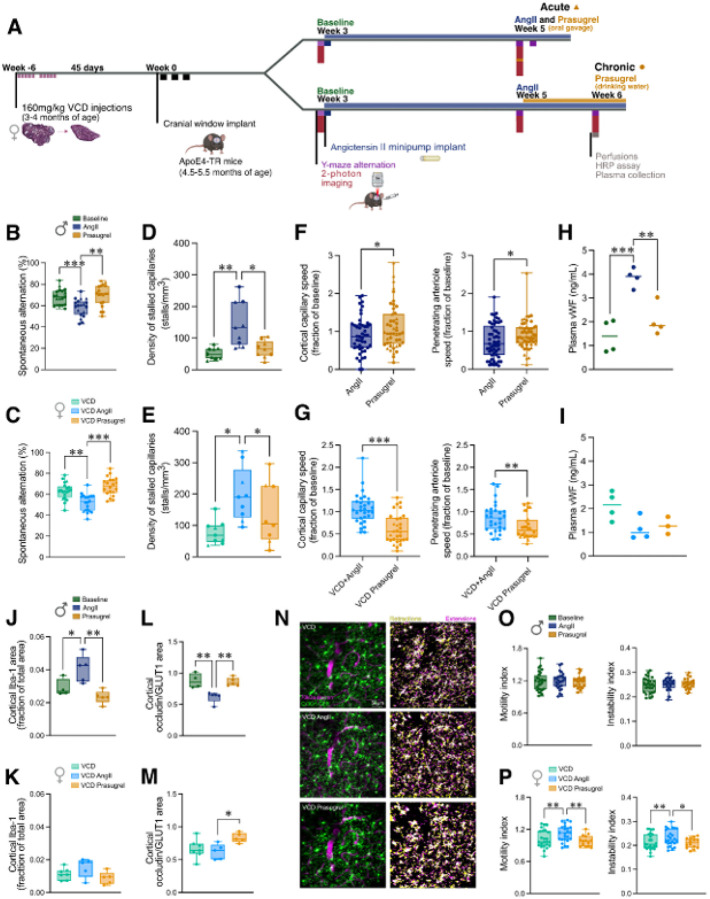
P2Y12 receptor inhibition with prasugrel improves memory function in ApoE4-TR mice, with sex-dependent effects on cerebrovascular function and neuroinflammation. (**A**) ApoE4-TR males and VCD-females underwent longitudinal assessments at baseline, after 2 weeks of angiotensin II (AngII), and after post-acute (▲) or 7-day chronic (●) prasugrel treatment. For in vivo imaging outcome measures post-acute data was taken starting 30 min after prasugrel treatment, while for behavioral outcome measures it was about 48 hours afterward. Y-maze assay in males (**B**), and VCD-females (**C**). Capillary stall incidence in males (**D**) and VCD-females (**E**). Cortical capillary and penetrating arteriole flow speeds after hypertension and prasugrel, expressed as a fraction of baseline, in males (**F**) and VCD-females (**G**). Plasma von Willebrand factor (vWF) by ELISA in males (**H**) and VCD-females (**I**). Immunohistochemistry of cortical Iba1+ in males (**J**) and VCD-females (**K**), and Occludin/GLUT1 ratio in males (**L**) and VCD-females (**M**). (**N**) 2PEF imaging of microglial process movement over 20 minutes, with retractions (yellow), extensions (magenta), and stable pixels (white). Microglia process motility and instability in males (**O**) and VCD-females (**P**).

Since we primarily observed hypertension-induced deficits in ApoE4-TR mice, we decided to utilize only ApoE4-TR mice moving forward. Motivated by both ApoE4 genotype and hypertension being associated with increased procoagulant status^42–45^, we hypothesized that the capillary stalling in this model may be mediated by endothelial and RBC interactions with activated platelets, facilitating RBC arrest. We thus used the anti-platelet agent, prasugrel, a P2Y12 receptor inhibitor that has not been reported to lower blood pressure. We evaluated both acute (~30 min (CBF) or 48h (behavior) after a single oral gavage of 10 mg/kg) and chronic (administered through drinking water, ~10 mg/kg/day for 7 consecutive days) treatment with this inhibitor (Fig. 4A). Prasugrel treatment did not attenuate the hypertension in ApoE4-TR mice treated with AngII (Supplementary fig. 13), but improved spatial working memory in both males (Fig. 4B, *p* < 0.01) and VCD-females (Fig. 4C, *p* < 0.001), as assayed by spontaneous alternation in the Y-maze. Regardless of treatment duration, prasugrel resulted in a 52% reduction in capillary stalling in males (65±9 vs. 144±24 stalls/mm^3^, *p* < 0.05, Fig. 4D). A more modest reduction in capillary stalling was observed in VCD-females (31% reduction, 138±40 vs. 198±29 stalls/mm^3^, *p* < 0.05, Fig. 4E). Intriguingly, prasugrel only improved CBF in males (Fig. 4F, showing matched vessels as a fraction of baseline flow), both in penetrating arterioles (20% increase, 8.6±0.7 vs. 10.3±0.5 mm/s, *p* < 0.05) and capillaries (23% increase, 3.2±0.3 vs. 3.9±0.4 mm/s, *p* < 0.05) without influencing vessel diameters (Supplementary fig. 14A-B). In contrast, we observed a ~19% decrease in CBF after prasugrel treatment in ApoE4-TR VCD-females in both penetrating arterioles (10.9±1.1 vs. 8.8±0.6 mm/s, *p* < 0.01) and capillaries (3.8±0.4 vs. 3.1±0.4 mm/s *p* <0.001), also without significant changes in vessel diameters (Fig. 4G).

Next, we examined the effects of chronic prasugrel treatment on peri-AOF or hypertension-induced neuropathological changes. We found trends towards decreased neuroinflammation as measured by Iba-1 (Fig. 4J and K, *p* < 0.01 in males, *p* = 0.08 in females) and GFAP (Supplementary fig. 4C-D, *p* = 0.08 in males, *p* = 0.12 in females). Regardless of sex, prasugrel significantly increased the levels of occludin coverage (Fig. 4L and M) and decreased MMP9 (Supplementary fig. 6C-D). With prasugrel treatment we observed some changes in circulating plasma cytokine levels, with a decrease in IGFBP-3 (*p* < 0.05) and leptin R (*p* < 0.01) exclusively in males, and an increase in PF4 (*p* < 0.01) in males and a trend toward an increase in PF4 in females (p= 0.13) (Supplementary fig. 8E-F). In plasma, the PC analysis showed prasugrel treated mice on the opposite side of the baseline condition from the AngII condition and along a similar axis, perhaps suggesting an overcorrection of some effects of hypertension (Supplementary fig. 8G). In cortical samples after prasugrel treatment, many cytokines were decreased with modest effect sizes, with ~2-fold more changes in ApoE4-TR males, as compared to VCD-females (Supplementary fig. 9). In the cortex, the PC analysis showed prasugrel-treated mice separating along a different direction (PC2) from the baseline and AngII treated groups separated along (PC1), suggesting prasugrel-specific changes in the brain rather than reversal of AngII effects (Supplementary fig. 9E).

To address whether prasugrel may be altering microglia behavior through P2Y12 inhibition, we quantified microglia motility in male and VCD-female Cx3cr1-ApoE3 and Cx3cr1-ApoE4 mice (Fig. 4N–P). In males, we observed no changes in microglial process dynamics neither with AngII for both genotypes nor after prasugrel treatment in ApoE4-TR mice (Fig. 4O and Supplementary Fig. 15A and C). However, in VCD-females we found that, at baseline, ApoE4-TR mice had lower motility, as compared to ApoE3-TR mice (Supplementary fig. 15B), and that Ang II increased and prasugrel then decreased process motility in ApoE4-TR mice (*p* < 0.01; Fig. 4P *left*), driven by changes in both extension (*p* < 0.01), and retraction (*p* < 0.05, Supplementary fig. 15D) of microglial processes. We also found a significant increase in the instability of processes with AngII (*p* < 0.01, Fig. 4P *right*), that was normalized with prasugrel (p<0.05, Fig. 4P *right*). Supporting the finding of more modest impacts of hypertension and prasugrel treatment on microglia in males, as compared to VCD-females, we also quantified microglia process morphology. In VCD-females, we found decreases in morphological complexity (fewer branches, junctions, and endpoints; shorter branch lengths) with prasugrel treatment, while in males there was no apparent effect (Supplementary fig. 16).

### Elucidating sexually dimorphic mechanisms of action of prasugrel

**Figure 5:**
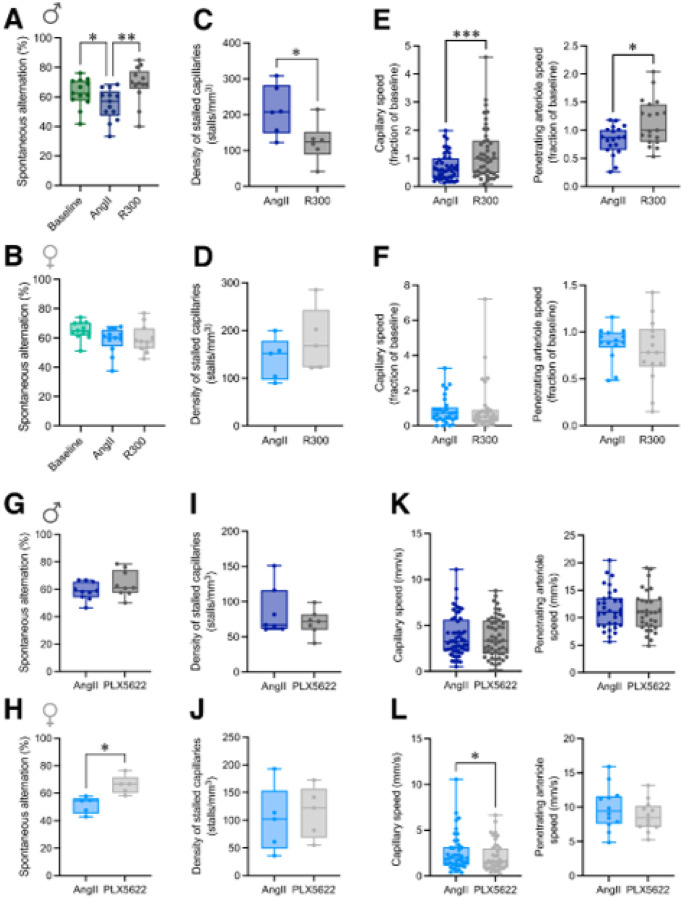
Platelet and microglia depletion have sexually-dimorphic effects on spatial working memory and blood flow in ApoE4-TR mice. Spontaneous alternation Y-maze scores in males (**A**) and VCD-females (**B**) 24 h following platelet depletion by R300, or after microglia depletion by PLX5622 (7 days) in males (**G**) and VCD-females (**H**). Quantification of capillary stalls in males (**C**) and VCD-females (**D**) following R300, or after PLX5622 in males (**I**), and VCD-females (**J**). (**E**) Capillary *(left)* and penetrating arteriole (PA) *(right)* flow speeds, expressed as a fraction of baseline, in males (**E**) and in VCD-females (**F**) after R300. Capillary (*left*) and PA (*right*) flow speeds before and after PLX5622 in males (K) and VCD-females (**L**).

In an attempt to gain insight into how prasugrel was mediating beneficial memory effects, two potential targets of P2Y12 inhibition were further investigated: platelets and microglia. First, while investigating platelets as a likely primary target of prasugrel, we used antibody-mediated platelet depletion in ApoE4-TR mice to determine whether loss of circulating platelets was sufficient to reverse hypertension-induced deficits. In males at 24 hours after platelet depletion, performance on spatial working memory was improved (Fig. 5A, *p* < 0.01), correlated with a reduced incidence of capillary stalls (Fig. 5C, *p* < 0.05), as well as increased capillary (Fig. 5E *left*, 1.99±0.28 vs. 3.03±0.43 mm/s, *p* < 0.001) and penetrating arteriole blood flow (Fig. 5E *right*,6.81±0.67 vs. 9.80±0.91 mm/s, *p* < 0.05). In stark contrast, platelet depletion in hypertensive VCD-female ApoE4-TR mice did not improve memory performance (Fig. 5B), reduce the incidence of capillary stalls (Fig. 5D), nor increase cerebral blood flow (Fig. 5F).

Supporting the idea that prasugrel is acting through platelet inhibition in males, but potentially not in females, we observed a clear sex difference in levels of circulating von Willebrand factor (vWF), which is correlated with endothelial activation and a prothrombotic state^46^. In males, we observed an almost four-fold elevation of vWF with hypertension only in ApoE4-TR mice (Fig. 4H, P < 0.001), with no significant elevation in ApoE3 mice (Supplementary fig. 17A). Treatment with prasugrel normalized vWF levels in ApoE4 males (*p* < 0.01, Fig. 4H). In contrast, in VCD-female mice there were no significant changes observed in vWF levels with hypertension or prasugrel treatment with either ApoE4 (Fig. 4I) or ApoE3 genotype (Supplementary fig. 17B).

To examine whether changes in microglia function may be involved in the beneficial effects of prasugrel treatment, we performed a depletion experiment where after 2 weeks of AngII, mice were placed on a PLX5622 diet for 7 days to achieve ~90% depletion of microglia (Supplementary fig. 18). In males, there was no behavioral improvement (Fig. 5G), capillary stalling rates did not change (Fig. 5I), nor were capillary and penetrating arteriole flow speeds increased (Fig. 5K) despite a significant increase in capillary diameter in males (Supplementary fig. 19, *p* < 0.001). In contrast, we observed an improvement in spatial working memory in females (*p* < 0.05, Fig. 5H), which was not accompanied by decreases in capillary stalling (Fig. 5J) nor increases in CBF (Fig. 5L).

## DISCUSSION

### Clinical relevance of risk-factor based mouse model of memory impairment

**Figure 6:**
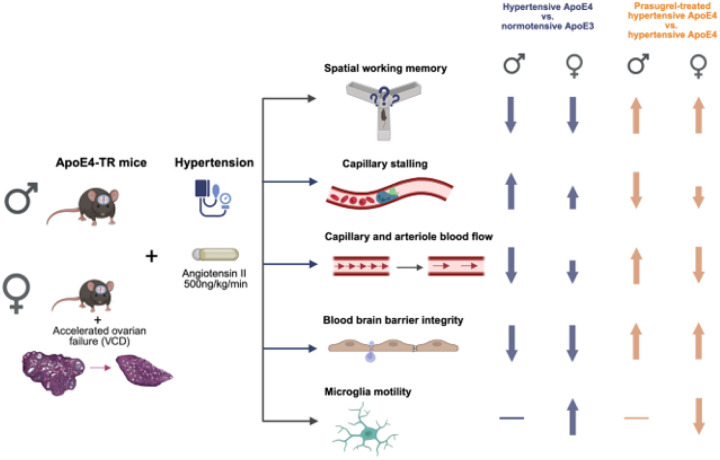
Summary figure of main results. Schematic showing the risk factors assessed on the left (ApoE4 genotype, hypertension, and perimenopause in female mice), with the primary outcome measures depicted and briefly described in the center. The blue arrows on the right illustrate the changes in these outcomes for hypertensive ApoE4 mice, as compared to normotensive ApoE3 mice, keeping males and females separate. The orange arrows illustrate the changes associated with prasugrel treatment in hypertensive ApoE4 mice. BioRender^™^ was used to make this schematic.

Studying the impact of risk factors for late onset dementia, including ApoE4, hypertension, female sex, and menopause is vital for understanding early triggers of cognitive decline. Individuals diagnosed with AD are 50% more likely than cognitively normal individuals to present with hypertension and are also more likely to be carriers of the ε4 allele of ApoE and be menopausal women^12,19,47^. Our study integrated these three prevalent ADRD risk factors into a novel mouse model. We showed that spatial working memory is significantly impaired only when hypertension co-occurred with ApoE4 genotype in mice (Figure 1), findings that parallel those in human studies. Independent of classic AD pathology, having the ε4 allele and/or hypertension contributes to a more rapid decline in cognitive function^48,49^. In a longitudinal study of middle-aged individuals from the Framingham Offspring study, higher pulse pressure was associated with worse visuospatial organization exclusively in ApoE4 carriers at a 5–7-year follow-up^23^. Additionally, in individuals deemed cognitively intact, episodic memory was uniquely impaired in ApoE4, but not ApoE3, carriers with mild blood pressure elevation^22^. In ApoE4-TR mice fed a high fat diet, Pontifex et al. similarly found that cognitive deficits were exacerbated compared to ApoE3-TR mice on a high fat diet or ApoE4-TR mice fed regular chow.^50^ While ApoE3-TR mice were impaired on Y-maze alternation and novel object recognition after bilateral carotid artery stenosis, ApoE4-TR mice performed significantly worse^51^. Collectively, these data suggest that there is a combined impact of ApoE4 and vascular risk factors on cognition, with a greater cognitive vulnerability in ApoE4 carriers to additional insults, such as obesity, carotid artery stenosis, or hypertension. Understanding how these risk factors mechanistically contribute to cognitive impairment is essential for effective, early intervention. In our work, we found that spatial working memory was rescued in both males and peri-AOF females with prasugrel treatment, and we uncovered sexual dimorphisms in the potential mechanisms contributing to memory function improvement, with rescue correlating with anti-platelet effects in males and with altered microglia phenotype in females (Figure 4). Furthermore, platelet depletion largely recapitulated the effects of prasugrel in males, but not females, while microglia depletion recapitulated the effects of prasugrel in females, but not males (Figure 5). These relationships are summarized in Figure 6.

### Brain blood flow alterations with ApoE4, hypertension, and AOF

We found that many measures of CBF were lower in ApoE4 mice, as compared to ApoE3 mice, and that hypertension consistently drove further decreases in CBF in ApoE4 mice (Figure 3). This finding is consistent with the larger blood flow decrease in the WM of male ApoE4-TR mice that was observed after bilateral carotid artery stenosis, as compared to ApoE3-TR mice^51^. CBF changes associated with ApoE4 genotype in humans are complex and vary with age, with increased CBF in some brain regions in young adulthood (early 20’s), but with pronounced decreases (~44%) in older adults (early 70’s), relative to ApoE3 carriers that remain more stable (~8% increase)^52^. In the Chinese Imaging, Biomarkers and Lifestyle study, the effect of ApoE genotype on cognition was found to be partly mediated by decreased CBF^53^. This correlation, however, was weakened if systolic blood pressure was adjusted for, perhaps reflecting both the known higher incidence of hypertension in ApoE4 carriers^18^, as well as the possibility for hypertension to exert a more deleterious influence in ApoE4 carriers^53^. Indeed, the reduced resting cerebral perfusion and impaired neurovascular coupling found in ApoE carriers likely makes them more sensitive to additional vascular insults, such as hypertension (present study) or carotid stenosis^51,54^.

Few studies have explored the role of CBF alterations during perimenopause or menopause on cognitive impairment. A meta-analysis from 2021 analyzing nine studies suggested a trend toward decreased CBF due to menopause but primarily concluded that most studies were underpowered and menopause reporting criteria were inconsistent, making definitive conclusions difficult^55^. A recent cross-sectional study in pre-, peri-, and post-menopausal women and age-matched men found that peri- and post-menopausal women have CBF levels comparable to age-matched men, while CBF was higher in pre-menopausal women^15^. Cerebrovascular reactivity may also be impacted by menopause, with one study indicating that despite normal performance on a Stroop task, perimenopausal women had a more variable neurovascular coupling response that depended on estrogen levels^56^. Because estrogen partly protects against cardiovascular and cerebrovascular dysfunction^57^, the decline in estrogen levels at menopause also increases the prevalence of vascular risk factors linked to dementia in women^12,28^. Cerebrovascular dysfunction may also be more impactful in older women than men, with one study showing that higher vascular risk scores were associated with lower cognitive performance in post-menopausal women, as compared to age-matched men^58^. Interestingly, while we found hypertensive ApoE4-dependent impairment of CBF in both sexes, these alterations were more consistent in males, with more variable and weaker impacts in peri-AOF females (Figure 3). Fluctuations in estradiol levels during peri-AOF that were not controlled for, due to the difficulty in measuring this longitudinally in mice, may have contributed to the variability observed in the females. Important extensions of our work include analyzing the impact at a menopause-like state using VCD-female mice at later timepoints when estradiol fluctuations have subsided. Finally, we note that while modeling perimenopause was essential to assess the impact of hypertension on female mice in this study due to estrogen’s protective effects against AngII-stimulated hypertension^59,60^, not all aspects of cerebrovascular function are impacted by menopause. For example, in a mouse model of carotid artery stenosis, VCD-induced AOF was not associated with more severe CBF deficits^61^.

### Platelet and inflammatory cell contributions to capillary stalling

While we observed an increase in capillary stalling due to hypertension in both male and peri-AOF female ApoE4 mice, this elevation was clearer in males (Figure 3). In addition, the cellular composition found within the stalled segments differed somewhat between males and females, with stalls containing RBCs dominating in males and leukocyte-mediated stalls dominating in females. Our and others’ prior work has uncovered a variety of causes of capillary stalling in mouse models of different underlying pathologies. Mouse models of polycythemia vera had an increase in stalls that only contained RBCs; essential thrombocythemia led to elevated stalls containing platelet aggregates^62^ as did a mouse model of type 1 diabetes^35^; in APP/PS1, 5XFAD, and APP^NL-G-F^ mouse models of AD stalls were caused by the arrest of neutrophils in capillaries^33,34^; neutrophil arrest has also been shown to contribute to decreased penumbral blood flow after a stroke^63^. Taken together these data suggest that capillary flow is precarious due to the single file flow of blood cells, enabling different upstream pathological processes to facilitate the arrest of different formed elements of the blood to cause a capillary stall. These diverse cellular causes of capillary stalling can, in turn, cause significant decreases in CBF that may be, at least in part, responsible for a decline in cognitive function.

In hypertensive ApoE4 males, but not females, we found increased plasma levels of vWF, suggesting a prothrombotic state, which has been associated with both ApoE4 genotype and hypertension in patients^42–45^. Other researchers found that endothelial levels of vWF were decreased in male mice expressing ApoE4 peripherally^64^, likely due to vWF shedding from endothelial cells, and thus consistent with the increased plasma levels we found. Interestingly, vWF has been proposed as a bridge connecting hemostatic and inflammatory pathways, so the increase in vWF levels identified here could increase adhesion and recruitment of platelets and other blood cells to the endothelium^65^, thus increasing capillary stalling. Indeed, altered platelet structure and function have been found in several neurodegenerative diseases, with increased platelet reactivity linked to increased disease severity^66^. In a mouse model of type 1 diabetes, treatment with anti-IL-10 reduced RBC-mediated stalls, and this correlated with downregulation of platelet activation pathways^35^, supporting a potential role for platelets in RBC-containing stalls. We thus hypothesized that activated platelets may facilitate RBC arrest and underlie the increased capillary stalling we observed in male hypertensive ApoE4 mice. Importantly, we note that our method of rhodamine 6G-based labeling of platelets is likely only sensitive enough to visualize platelet aggregates and not individual platelets that are possibly decorating stalled RBCs. Motivated by this idea, we treated hypertensive ApoE4 mice with an antiplatelet therapy, prasugrel, which was approved by the FDA in 2009 for reducing the risk of thrombotic cardiovascular events^67^, but may also be protective in neurodegenerative disease,^66^ especially vascular dementia^68^. In hypertensive ApoE4-TR males, we found improved memory performance that we attribute primarily to prasugrel’s antiplatelet effects. Stalls were reduced and CBF was increased both within an hour of prasugrel treatment, as well as after one week of treatment in the continued presence of AngII infusion and hypertension (Figure 4), which we would expect to drive further decreases in CBF. In addition, vWF levels were normalized, suggesting a reduced pro-thrombotic state. In animal models of ischemic injury, the effects of prasugrel have been correlated with improved blood flow to affected organs and tissues^69–72^. Prasugrel improves blood flow by reducing the likelihood of thrombotic events to occur by decreasing platelet activation and aggregation, reducing platelet-leukocyte interactions^73^, decreasing blood viscosity^74^, and, in our data, reducing the incidence of capillary stalling (Figure 4). Further supporting the idea that the impact of prasugrel in males is through platelet inhibition, we found that platelet depletion similarly reduced stalling, increased CBF, and led to improved memory function in hypertensive male ApoE4 mice (Figure 5). The lower efficacy of prasugrel in reducing the incidence of capillary stalls in hypertensive ApoE4 VCD-female mice is consistent with the lack of vWF elevation, suggesting a lower pro-thrombotic state, and leukocyte arrest being the dominant cause for stalls in this group. The striking further decrease in CBF in ApoE4 VCD-female mice after prasugrel treatment warrants further investigation, and emphasizes the need for AOF models in the study of neurodegeneration.

### BBB compromise with hypertension and ApoE4, and restoration with prasugrel

We found the largest compromises of BBB integrity in male and VCD-female mice with the combination of hypertension and ApoE4 genotype (Figure 2). Such BBB compromise may be linked to the capillary stalling we observed, as loss of tight junction proteins could expose basement membrane and increase the likelihood of circulating cells and platelets to adhere^75^. Paralleling our findings, other researchers have found that expression of peripheral ApoE4 in male mice compromises the integrity of the basement membrane by decreasing collagen IV and increased vessel-associated gliosis in the brain^64^. Interestingly, when ApoE3 mice were exposed to plasma from male ApoE4 mice there was a decrease in BBB integrity without evidence of cognitive impairment^64^. In contrast, other researchers found no differences in occludin expression between ApoE4-TR and ApoE3-TR mice, even at 12 months of age^76^. Hippocampal BBB leakage was also found in human ApoE4 carriers, in the absence of amyloid or tau pathology, and was worse in individuals with mild cognitive impairment^77^. Similarly, hypertension has been associated with decreased BBB integrity, which was mitigated by antihypertensive medication.^78^ Collectively, these data suggest subtle changes in BBB integrity associated with ApoE4, which may be exacerbated by hypertension.

After prasugrel treatment, we observed an increase in occludin and a decrease in vascular MMP9 in both sexes, indicative of a tighter BBB. In agreement with these findings, in an *in vitro* study using a lipopolysaccharide-induced inflammatory injury in human pulmonary microvascular endothelial cells, treatment with the P2Y12 receptor inhibitors, ticagrelor or clopidogrel, increased levels of tight junction proteins, including VE-cadherin, ZO-1, claudin 5, and occludin^79^. Such barrier tightening after prasugrel may be due to increased intracellular cyclic adenosine monophosphate levels, which is known to strengthen endothelial cell barrier properties^80–82^. P2Y12 inhibition, however, has been found to play both protective and pathological roles in BBB permeability, in part due to the impact on microglia behavior, depending on the nature of and time since the vascular injury^83,84^.

### Platelet-independent effects of prasugrel that may account for sexual dimorphisms

We found a mild neuroinflammatory phenotype associated with hypertension and/or ApoE4 genotype, with some variation between sexes (Figure 2). In cortical samples from both hypertensive ApoE4 males and VCD-females, we found elevated IL-1α, a cytokine indicative of early, mild inflammation that serves as an alarm to immune cells, but no elevation of IL-1β, (associated with more advanced stages of neuroinflammation^85^. In agreement with this finding, hypertension has been associated with chronic low-grade peripheral- and neuro-inflammation in patients^86,87^. Such inflammation has been associated with shortened latency for AD onset and increased medial temporal lobe atrophy in ApoE4 carriers, as compared to both ApoE3 carriers, and ApoE4 carriers without low-grade inflammation^88^. Several studies have shown that hypertensive stimuli, including AngII, elicit a response from microglia, implicating them in the early neuroinflammatory response to vascular dysfunction^89^. We found clear microglia responses to hypertension in ApoE4 VCD-female mice, with changes in both cell morphology and the movement of processes. In male ApoE4 mice, while we found an increase in the area covered by microglia, there were fewer changes in cell morphology and process movement. Sex differences in the expression of different AngII receptors^90^ may have contributed to the differences in microglia response to hypertension we found. We also found a larger number of cortical cytokines that increased by hypertension together with larger increases in VCD-females, as compared to males. Collectively, these data suggest hypertension drives a stronger inflammatory phenotype in the cortex of ApoE4 VCD-female mice, as compared to ApoE4 males.

We did not find a straightforward relationship between capillary stalling, CBF, and memory performance in peri-AOF ApoE4-TR female mice. Instead, while prasugrel treatment robustly rescued spatial working memory, as in males, it was associated with a modest and variable reduction in capillary stalls and with decreased, rather than increased, CBF (Figure 4). Several factors may contribute to this sexual dimorphism in response to prasugrel. First, there are known sex differences in the response to antiplatelet therapies. Women have higher baseline platelet reactivity in response to agonists compared to men, even while on antiplatelet therapies, and have an increased propensity to form platelet-leukocyte aggregates^91–93^. Platelet reactivity is affected by estrogen levels, so women are in a more hypercoagulable state after menopause^94^, suggesting an increased dose of antiplatelet therapy may be needed to provide adequate protection. The continued CBF decrease in females after prasugrel treatment is paradoxical. While continued infusion of AngII and the effects of the resulting hypertension would be expected to cause additional CBF decreases in the mice treated for one week with prasugrel, we also found that a single oral gavage of prasugrel decreased CBF within an hour, too fast for ongoing effects of hypertension to manifest. One possible explanation is the stronger impact we observed of P2Y12 inhibition on microglia in females. Recent work has implicated microglia, acting via purinergic receptors, in the regulation of vascular tone and neurovascular coupling responses^95–97^. These studies show that P2Y12 receptors on microglia are essential for maintaining CBF and regulating responses to stimuli. We found prasugrel treatment-dependent changes in microglia behavior only in ApoE4 VCD-female mice, suggesting the possibility of sex-dependent changes in the impact of microglia on CBF. The dominant cause of capillary stalling in the hypertensive ApoE4 VCD-female mice was arrested leukocytes, and an important follow up to this study would be to evaluate changes in CBF and memory performance when the incidence of these leukocyte-mediated stalls was decreased.

In females, we found that cognitive rescue by prasugrel was independent of CBF recovery. Based on predicted physiochemical properties, prasugrel is expected to cross the BBB effectively^98^, and the increased BBB permeability we found in hypertensive ApoE4 mice would further enable prasugrel penetration into the brain. Following prasugrel treatment we found a decrease in microglia process motility and ramification exclusively in hypertensive peri-AOF ApoE4 female mice. This larger response to prasugrel treatment may be due to higher P2Y12 expression in microglia in females^99^, which could make them more sensitive to P2Y12 inhibition by prasugrel. There are prior reports suggesting P2Y12 inhibition can be neuroprotective, largely by reducing microglia-mediated inflammation and recruitment in both stroke and later stage AD^100^. For example, using bilateral carotid artery stenosis in mice pre-treated with clopidogrel and using oxygen-glucose deprivation in a neuron-astrocyte-microglia co-culture where microglia P2Y12 was knocked down using siRNA, Webster et al. found that P2Y12 inhibition was protective against brain ischemia as measured by neuronal loss^101^. Our attribution of the cognitive recovery after prasugrel treatment in female mice to changes in microglia behavior is further supported by our finding that microglia depletion by PLX5622 over 7 days also resulted in improved memory performance exclusively in females, again without increasing CBF.

### Implications

In this study, we established a translationally relevant mouse model that integrates three major risk factors for ADRD — ApoE4 genotype, hypertension, and biological sex with peri-AOF — to investigate the early mechanisms driving cognitive decline. We found that these risk factors interact to impair spatial working memory through cerebrovascular dysfunction and neuroinflammation, in the absence of amyloid or tau pathology. Notably, we identified sex-specific mechanisms underlying these deficits: in males, platelet-mediated capillary stalling and reduced cerebral blood flow were key contributors, while in peri-AOF females, microglia state and immune signaling seemed to play a more dominant role. Treatment with the P2Y12 inhibitor prasugrel rescued memory performance in both sexes but did so via distinct pathways – restoring CBF in males while modulating microglia phenotype in females. The consistency between our finding of a normalization of plasma vWF with prasugrel treatment, together with capillary stall reduction, CBF increase, and memory improvement following either prasugrel treatment or platelet depletion in hypertensive ApoE4 male mice suggests targeting platelets may be a promising prospect in men with a similar risk profile. The more complex physiological impacts of prasugrel in the female mice warrants further investigation to more deeply understand mechanisms contributing to memory improvement. Overall, our findings reinforce the notion that therapeutic strategies targeting cerebrovascular-related cognitive decline may be especially effective when tailored by sex and genetic background, particularly for ApoE4 carriers. This could allow for earlier preventative strategies, with more targeted approaches based on risk profiles. Our work also underscores the need to incorporate female models that reflect transitional hormonal states such as perimenopause into studies of neurodegenerative disease.

## Supplementary Files

This is a list of supplementary files associated with this preprint. Click to download.

- SupplementaryMaterial.docx

## Data Availability

All data are available in the main text or the supplementary materials. Any raw data will be made available upon reasonable request.
